# Reactivation of Chronic Hepatitis C as a Potential Trigger for Guillain-Barré Syndrome

**DOI:** 10.7759/cureus.5244

**Published:** 2019-07-26

**Authors:** Ogochukwu Molokwu, Brittany M Young, Manjit Singh, Krishe Menezes, Raza Mian

**Affiliations:** 1 Medicine, University of California San Francisco Fresno School of Medicine, Fresno, USA; 2 Internal Medicine, University of California San Francisco Fresno School of Medicine, Fresno, USA

**Keywords:** gbs, hepatitis c, reactivation, hcv

## Abstract

A 74-year-old man with a past medical history of chronic low back pain presented with two to three weeks of progressive weakness starting in the lower extremities and then spreading to the upper extremities. Distal muscles were more affected than proximal muscles; weakness was accompanied by numbness and paresthesias. There was no preceding acute viral, respiratory, or gastrointestinal illness. Initial workup revealed hepatitis C antibody reactivity, and cerebrospinal fluid (CSF) analysis showed albuminocytologic dissociation. MRI demonstrated multilevel degenerative changes and diffuse enhancement of the cauda equina nerve roots compatible with Guillain-Barré syndrome (GBS). Repeat testing confirmed ongoing hepatitis C infection with increasing quantitative hepatitis C virus (HCV) levels. This case illustrates an interesting presentation of GBS potentially triggered by hepatitis C reactivation. This is the first case, to our knowledge, with serologic evidence demonstrating acute hepatitis C reactivation concurrent with GBS which presented in the absence of immunomodulatory interferon treatment. The patient continues to recover with ongoing rehabilitation at the time of this case report.

## Introduction

Guillain-Barré syndrome (GBS) is an autoimmune neurologic condition characterized by a relatively acute presentation of areflexic paralysis often accompanied by albuminocytologic dissociation noted on cerebrospinal fluid analysis. While its exact etiology remains incompletely characterized, there is evidence that at least some subtypes of GBS result from an environmental trigger that leads to cross-reactivity and subsequent autoimmune damage to neurons [1]. Infectious agents have been the most commonly implicated triggers for this process. Approximately two-thirds of patients show evidence of an acute infection in the weeks preceding the onset of GBS with respiratory or gastrointestinal symptoms being most commonly reported [2,3]. Numerous pathogens have been catalogued as possible triggers for GBS. Campylobacter jejuni is the most frequently implicated organism, with Haemophilus influenzae, Mycoplasma pneumoniae, Salmonella species, varicella-zoster virus, cytomegalovirus, and Epstein-Barr virus also being identified as triggering agents for GBS [2]. However, in most cases no specific causative organism can be definitively identified [1].

Although not as commonly implicated, infectious hepatitis has also been identified as a possible trigger for GBS [4,5]. While numerous cases of GBS preceded by hepatitis A or hepatitis E infection are documented in the literature, cases of GBS in the setting of recent or ongoing infection with hepatitis B or hepatitis C virus (HCV) are less commonly described. In this report, we describe a case of GBS observed in the context of acute reactivation of chronic HCV infection in the absence of any preceding respiratory, gastrointestinal, or other known acute viral infection.

## Case presentation

A 74-year-old white Hispanic male with a history of chronic low back pain in the setting of lumbar spinal stenosis status post L5-S1 posterior lumbar interbody fusion about two and half years ago, and subsequent revision with L4-L5-S1 interbody fusion 19 months ago, hypertension, hyperlipidemia, and tobacco abuse initially presented to an outpatient neurology clinic with 2-3 weeks of progressive weakness. The weakness was described as starting in the bilateral lower extremities and progressing to include the bilateral upper extremities. Distal muscles were more affected than proximal muscles in all four extremities. This weakness was accompanied by numbness and paresthesias, along with pain in his bilateral wrists and hands. The patient consistently denied any preceding viral, respiratory, or gastrointestinal illness. He had no known history of tick bites. Initial outpatient workup revealed normal thyroid function tests, unremarkable electrolytes, an unremarkable liver function panel (Table 1), and negative HIV. The patient’s anti-nuclear antibody testing was reactive, but the reflex panel (including anti-Ro/SSA and anti-La/SSB antibodies, anti-SCL-70, anti-JO-1, anti-smith, anti-double stranded DNA, anti-RNP, and anti-chromatin antibodies) was negative. Hepatitis C antibody testing was reactive, with quantitative hepatitis C virus (HCV) RNA > 3,000,000 (Table 1). Electrophysiologic studies of the upper extremities demonstrated evidence of both motor and sensory nerve involvement with chronic neuropathy motor unit action potentials suggestive of chronic motor sensory polyneuropathy with predominantly demyelinating and axonal features.

**Table 1:** Liver function serum markers and hepatitis C viral loads over time U: Units; L: liter; mg: milligram; dL: deciliter; IU: International units; AST: Aspartate aminotransferase; ALT: Alanine aminotransferase; INR: International normalized ratio; HCV RNA PCR: Hepatitis C virus ribonucleic acid polymerase chain reaction.

Laboratory test	Outpatient evaluation (Two weeks prior to admission)	Hospital day 1 (labs on admission)	Hospital day 4	Hospital day 27 (day of discharge)	4.5 months post-discharge
AST (U/L)	29	30	31	41	38
ALT (U/L)	40	39	45	38	49
Alkaline phosphatase (U/L)	Not measured	89	92	74	85
Total bilirubin (mg/dL)	1.1	0.7	1.5	1.1	0.9
INR	Not measured	1.1	0.9	Not measured	1.0
Hepatitis C antibody	Reactive	Reactive	Reactive	Not evaluated	Reactive
Quantitative HCV RNA PCR (IU/mL)	3,800,000	10,100,000	24,200,000	Not measured	7,480,000
Quantitative HCV RNA PCR (Log IU/mL)	6.58	7.00	7.38	Not measured	6.87

Two weeks later the patient was admitted for further inpatient workup, by which time he had lost the ability to use his hands or stand unassisted. The patient had become dependent on others for all his activities of daily living (ADL). The patient’s medications consist of diazepam, gabapentin, hydrocodone-acetaminophen, budesonide-formoterol, montelukast, and albuterol. Cerebrospinal fluid analysis revealed marked albuminocytologic dissociation (Table 2). MRI demonstrated multilevel degenerative changes and moderate stenosis of the spinal canal without acute cervical or thoracic abnormalities and anterior and posterior spinal fusions at L4-L5 and L5-S1. Diffuse enhancement of the cauda equina nerve roots compatible with Guillain-Barré syndrome was also reported. Testing for GQ1b antibody was negative (titer <1:100). HCV genotyping confirmed ongoing HCV genotype 2 infection with an increasing viral load (repeat quantitative HCV RNA PCR > 10 million, then >24 million). Serologic markers of liver function, including AST, ALT, alkaline phosphatase, total bilirubin and INR were generally within normal range (Table 1). Abdominal ultrasound revealed hepatomegaly with heterogeneity of the hepatic parenchyma and slight surface nodularity of the liver suggestive of cirrhosis, borderline splenomegaly, left renal cysts, and an incidental 3.2 cm distal aortic saccular aneurysm.

**Table 2:** Cerebrospinal fluid studies obtained on hospital day 2 demonstrating albuminocytologic dissociation CSF: Cerebrospinal fluid; RBC: Red blood cells; WBC: White blood cells; mm: millimeter; mg: milligram; dL: deciliter.

CSF Test	Value	Units
RBC	0	cells/mm^3^
WBC	1	cells/mm^3^
Cells counted	54	cells
Lymphocytes	76	%
Monocytes	24	%
Glucose	89	mg/dL
Protein	626	mg/dL

Serologic studies for evidence of ongoing or prior infection with hepatitis B and E viruses were negative, and hepatitis A IgG was reactive with negative hepatitis A IgM. In addition, polymerase chain reaction (PCR) studies of the cerebrospinal fluid (CSF) were negative for detection of cytomegalovirus, varicella zoster virus, herpes simplex viruses 1 and 2, human herpes virus 6, human echovirus, and enterovirus. Epstein-Barr virus was not tested. Stool studies for Campylobacter jejuni infection were also negative. Given his relative lack of risk factors for hepatitis C exposure (no history of IV drug use, no history of blood transfusion, no sexual relationships with known Hep C carriers), he is believed to have contracted hepatitis C years earlier from contaminated tattoo equipment.

The patient received a series of five plasma exchange treatments followed by five days of daily IVIG infusions. He remained stable throughout his hospital admission but showed only mild improvement by the time he was discharged to a skilled nursing facility for ongoing care and rehabilitation. Three months after hospital discharge the patient had improved upper and lower limb strength, proximally more than distally, following completion of inpatient rehabilitation therapy. He continued to have fasciculations in his bilateral upper limbs and areflexia of all four extremities at three-month follow-up. The patient had liver function tests and quantitative hepatitis C testing repeated 4.5 months after discharge, which showed a reduced viral load of HCV RNA < 7.5 million as compared to levels documented during the patient’s acute GBS presentation (Table 1). The reduction in HCV viral load occurred prior to the initiation of any hepatitis C treatment, and although the underlying mechanism is unclear, transient improvement in patient's immune status is a likely factor. This reduction in viral load correlates with clinical improvement noted in this patient. The patient was treated for hepatitis C with sofosbuvir-velpatasvir, with HCV RNA viral load undetectable following completion of a three-month course of this therapy. The patient continues to make slow improvements in strength and independence with ongoing outpatient rehabilitation and follow up with his neurologists.

## Discussion

Each year GBS affects about one in every 100,000 people, comprising approximately 3000-6000 new cases in the United States each year [6]. Here we describe an interesting case of GBS which presented concurrently with the acute reactivation of a chronic hepatitis C infection. Although no specific mechanism has been fully characterized by which infection with HCV or any other exposure can precipitate GBS in humans, the association between GBS and preceding infection with various pathogens including Campylobacter jejuni, Mycoplasma pneumoniae, cytomegalovirus, Epstein-Barr virus, and hepatitis A and E has been well-documented [2,4-5]. A similar phenomenon has also been observed in patients with HIV, for whom GBS symptoms can present at any stage of infection [7].

As of 2016, approximately one percent of adults in the United States (i.e., nearly 2.4 million individuals) were infected with hepatitis C, an estimated half of which are unaware that they have been infected [6]. In cases of GBS presenting in association with viral hepatitis, the majority of documented cases involve an acute hepatitis regardless of which hepatitis virus is identified, with hepatitis A and hepatitis E infections being the most commonly implicated viral hepatitides [4-5]. Less frequently, GBS has been described in patients in whom chronic viral hepatitis infection status is either known or cannot be ruled out [8-10]. However, such cases describing GBS with concurrent hepatitis B or C infection frequently involve clinical features more suggestive of acute infection, administration of interferon therapy (a treatment that has been independently implicated as a potential trigger for GBS, or hypothesized acute reactivation of a chronic infection [9-12, 2]. There have also been multiple cases of GBS that presented in the context of “non-A non-B hepatitis,” some of which are suggestive of an acute infection, while others presented with normal liver function studies or evidence only of mild subclinical hepatitis [4, 13]. These reports predate the standardization of testing for HCV infection and thus may represent additional cases of GBS associated with acute or chronic infection with HCV.

While there are a few GBS cases in the literature that have presented in the context of documented hepatitis C, this is the first to our knowledge with serologic evidence demonstrating acute HCV reactivation concurrent with GBS symptoms [9-10,12]. In this case report, we describe a patient with a type of GBS in the context of HCV infection. The diagnosis of HCV was made during the initial workup, with no prior documentation of HCV diagnosis in patient’s medical records and no recollection of such diagnosis in the past by the patient or the patient’s family.

It is well known that HCV can trigger disorders that stem from dysfunction of the adaptive immune system such as cryoglobulinemia [14]. The primary mechanisms behind the development of GBS proposed thus far are also rooted in immunologic dysfunction [1]. Most patients with chronic untreated hepatitis C will have a stable long-term HCV-RNA viral load which varies within 0.5 log10 [15]. As suggested above, transient reactivation of HCV or enhanced HCV replication could be responsible for development of GBS in chronic hepatitis C patients [10]. We believe that reactivation of chronic hepatitis C is potentially responsible for precipitating GBS in our patient. Hepatitis C reactivation has been defined as an increase of HCV-RNA viral load greater than 1 log10 IU/mL from baseline [16-17]. In the case of our patient, there is clear serologic evidence of rapid and significant increase in HCV viral load. Although the patient's baseline HCV-RNA viral load is unknown, as HCV was first detected in this patient during workup after GBS symptoms had already presented, there was an 0.8 log10 increase in HCV-RNA viral load within a 17-day period noted during his clinical course. Although this increase does not meet the standard criteria of 1 log10 IU/mL from baseline, we believe it to be suggestive of hepatitis C reactivation as the patient's baseline pre-GBS HCV RNA level is unknown but was likely to have been lower than the initial level noted during GBS workup.

Despite chronic hepatitis infection with evidence of cirrhosis on imaging, the patient’s liver function tests (LFTs) remained largely within normal limits throughout his clinical course. This is not too surprising, as normal LFTs may be observed in patients with chronic hepatitis or cirrhosis, but it does further argue against an acute viral hepatitis. In a new-onset HCV infection or acute hepatitis, elevated liver function enzymes would have been expected. Reactivation of hepatitis C has been reported extensively in patients treated with chemotherapy or immunosuppressive therapy [16-18]. While the provoking factors for hepatitis C reactivation in this patient, who had not received any chemotherapy or immunosuppressive treatments, remain unclear, the patient's cirrhosis may have played a role. Given the alterations in both innate and acquired immunity seen in cirrhotic patients, cirrhosis, regardless of its etiology, is considered to be an immunocompromised state [19]. In one study, liver cirrhosis was significantly associated with enhanced HCV replication, which was attributed to altered immunity in the cirrhotic liver and abnormalities in B-cell function seen in cirrhotic patients [17]. As such, this patient’s cirrhosis may have facilitated this acute reactivation of his chronic hepatitis C infection.

It should be acknowledged that GBS in this patient could have been caused by an unidentified trigger with hepatitis C infection being only an incidental finding. Co-infection of hepatitis C with another GBS-associated pathogen such as Epstein-Barr virus, which was unfortunately not tested during the patient’s workup, is impossible to exclude at this time. However, the known association between hepatitis and GBS suggests that reactivation of chronic hepatitis C could be a plausible culprit. Being in an immunosuppressed state is also, itself, a recognized potential trigger for GBS [2]. Thus, it is possible that the patient’s cirrhosis and associated immune dysfunction was a trigger for both hepatitis C reactivation as well as GBS.

This patient received the appropriate treatments of plasma exchange and IVIG with only minimal symptom improvement over the course of his hospital stay. The patient also received appropriate therapy for his hepatitis C with reduction in viral load to undetectable levels in the months following hospital discharge. During this time frame, he has made slow, steady gains with both inpatient rehabilitation and ongoing outpatient rehabilitation, regaining the ability to walk using a walker and perform limited ADLs with his upper extremities. One similar situation has been described in the literature, in which a patient with GBS presenting in the context of acute on chronic hepatitis B infection achieved concurrent recovery from both hepatitis B infection and GBS symptoms following a course of Entecavir with concurrent IVIG treatment [8]. However, since symptoms of GBS tend to improve over the course of months to years in the majority of affected patients, it is impossible to establish the extent to which these improvements in the patient’s GBS symptoms might be attributable to resolution of the viral infection rather than being part of the natural course of the disease in this patient [20].

GBS can be a very debilitating condition that requires prompt recognition and treatment, and viral hepatitis is increasingly being recognized as a possible trigger for this disease. In light of the above, we suggest that reactivation of chronic hepatitis C should be considered as a possible underlying mechanism in patients with diagnosed GBS. Current guidelines do not discuss testing for viral hepatitis in patients with suspected or confirmed GBS. However, further testing in the affected population would allow for better characterization of the association HCV infection may have with GBS as has been demonstrated for other viral infections such as HIV [7]. We recommend HCV screening in patients suspected to have GBS with no known history of HCV. Similarly, we recommend HCV-RNA viral load to be assessed in patients with known chronic HCV infection as part of the initial GBS workup.

## Conclusions

This case illustrates an interesting presentation of GBS observed concurrently with hepatitis C reactivation. In patients suspected to have GBS, and with no clear preceding acute respiratory or gastrointestinal illness, testing for hepatitis C and other viral hepatitis should be included as part of the initial workup.
